# Supplementary material for: Regulation of NSL by TAF4A is critical for genome stability and quiescence of muscle stem cells
Source: Nat Commun. 2025 Sep 30;16:8726. doi: 10.1038/s41467-025-64402-1 (PMC12484798; doi:10.1038/s41467-025-64402-1)
Supplement: Supplementary file 1 — Supplementary Information [file 41467_2025_64402_MOESM1_ESM.pdf]

Supplementary Information

Regulation of NSL by TAF4A is critical for genome stability and quiescence of muscle stem cells

Angelina M. Georgieva[1], Krishna Sreenivasan[1], Dong Ding[1], Clementine Villeneuve[2], Sara A. Wickström[2], Stefan Günther[1], Carsten Kuenne[1], Ulrich Gärtner[3], Xinyue Guo[1], Yonggang Zhou[1], Xuejun Yuan[1]* and Thomas Braun[1, 4]*

[1]Department of Cardiac Development and Remodeling, Max Planck Institute for Heart and Lung Research, 61231, Bad Nauheim, Germany

[2] Department of Cell and Tissue Dynamics, Max Planck Institute for Molecular Biomedicine, 48149 Münster, Germany

[3] Institute for Anatomy and Cell Biology, University of Giessen, 35392, Giessen, Germany

[4] Division of Life Science, Center for Stem Cell Research, The Hong Kong University of Science and Technology, Hong Kong SAR, China.

*Corresponding authors:

Thomas Braun. Email: thomas.braun@mpi-bn.mpg.de

Xuejun Yuan. Email: xuejun.yuan@mpi-bn.mpg.de

Dept. of Cardiac Development and Remodeling

Max-Planck-Institute for Heart and Lung Research

Ludwigstr. 43, Bad Nauheim

Germany

Supplementary Information **Page**

Supplementary Information	Page
Supplementary Figure 1	3
Supplementary Figure 2	4
Supplementary Figure 3	5
Supplementary Figure 4	7
Supplementary Figure 5	9
Supplementary Figure 6	11
Supplementary Figure 7	13
Supplementary Table 1	14
Supplementary Table 2	15
Supplementary Table 3	16
Supplementary Table 4	17

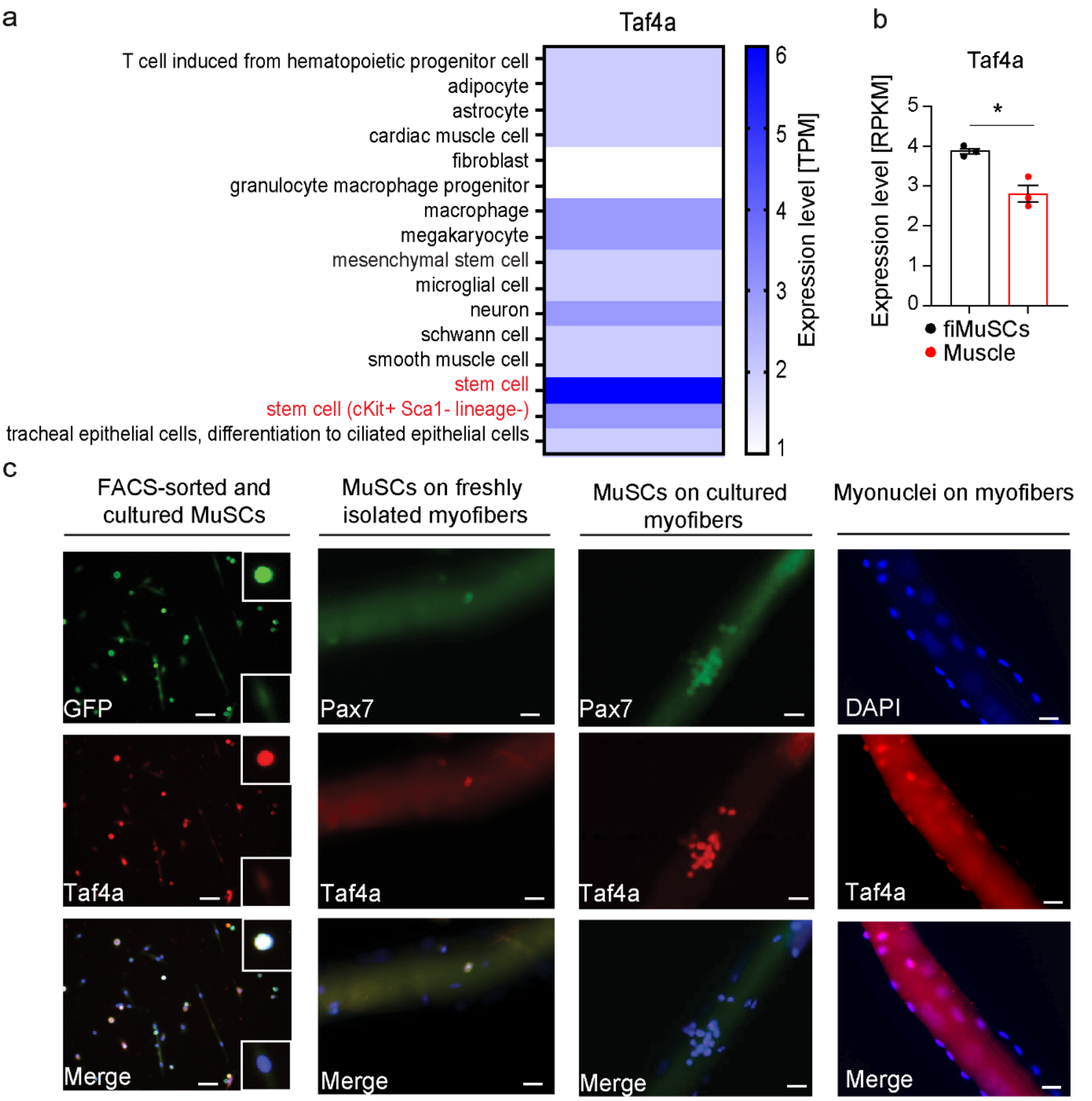

Supplementary Figure 1. ***Taf4a* is highly expressed in MuSCs but not in myofibers. a** *Taf4a* RNA-Seq CAGE (Cap Analysis of Gene Expression) expression analysis using data from the RIKEN FANTOM5 project. **b** *Taf4a* expression in freshly isolated MuSCs (fiMuSCs) and total muscle based on reads per exon kilobase per million (RPKM) values (unpaired two-tailed t-test: * p=0.0103, n=3). **c** Immunofluorescence staining of TAF4A in cultured MuSCs, 3 days after by FACS-based isolation of MuSCs using Pax7-zsgreen reporter mice (first row), in MuSCs found on 50 freshly isolated myofibers (second row), and in MuSCs found on 50 myofibers after culturing for 3 days (third and fourth panel), n=3. Scale bar: 20 µm. Data are presented as mean ± SEM. Source data are provided in the Source Data file.

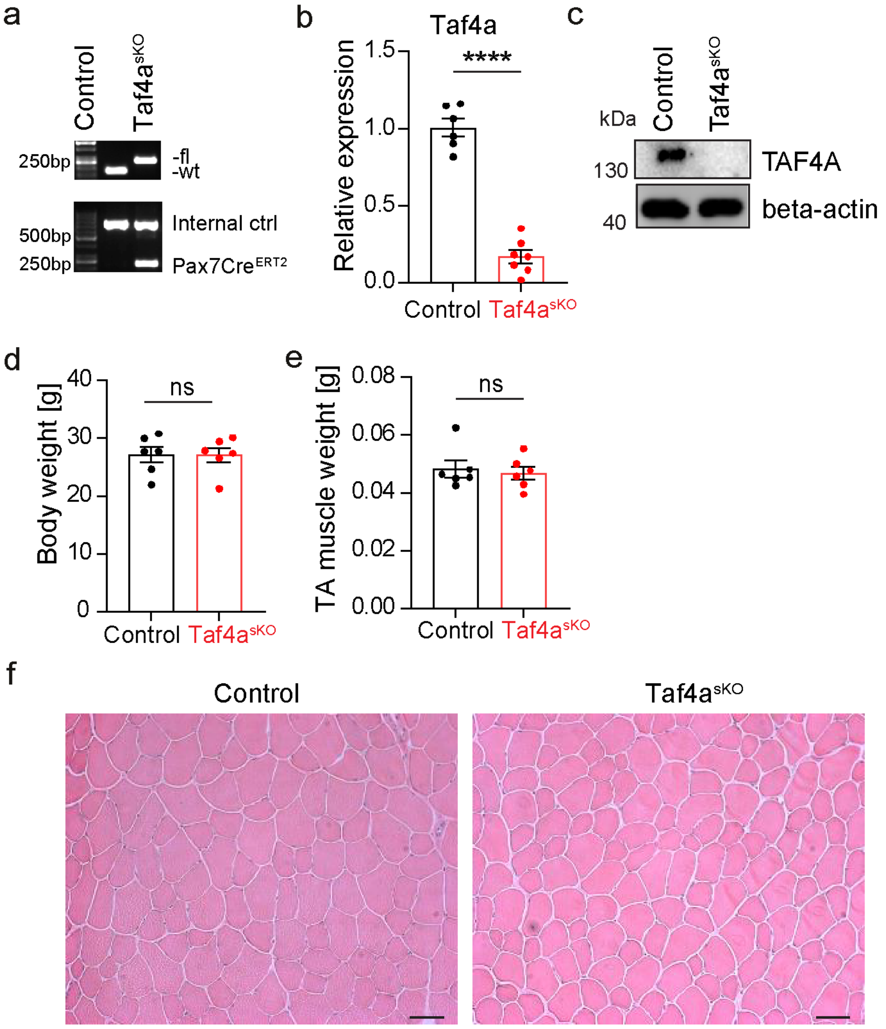

Supplementary Figure 2. MuSC-specific inactivation of *Taf4a* in adult mice does not alter skeletal muscle morphology under physiological conditions. a Representative genomic PCR analysis for detection of WT and deleted *Taf4a* alleles (upper panel). Detection of the presence of the *Cre* alelle by genomic PCR analysis using Pax7CRE^{ERT2} specific primer sets (bottom panel). **b** RT-qPCR analysis of *Taf4a* expression levels in freshly isolated control (n=6) and *Taf4a^{sKO}* (n=7) MuSCs. *m36b4* was used as reference gene (unpaired two-tailed t-test ****p <0.0001). **c** Western blot analysis of TAF4A levels in cultured MuSCs from control and *Taf4a^{sKO}* mice. Beta-actin was used as loading control (n=2). **d** Body weight of 21-weeks-old control and *Taf4a^{sKO}* mice, 14 days after tamoxifen treatment (unpaired two-tailed t-test: ns p > 0.05, n=6). **e** TA muscle weight of 21-weeks-old control and *Taf4a^{sKO}* mice (unpaired two-tailed t-test ns p>0.05, n=6). **f** H&E staining of TA muscle from control and *Taf4a^{sKO}* mice (n=3). Scale bar: 20µm. Data are presented as mean ± SEM. Source data are provided in the Source Data file.

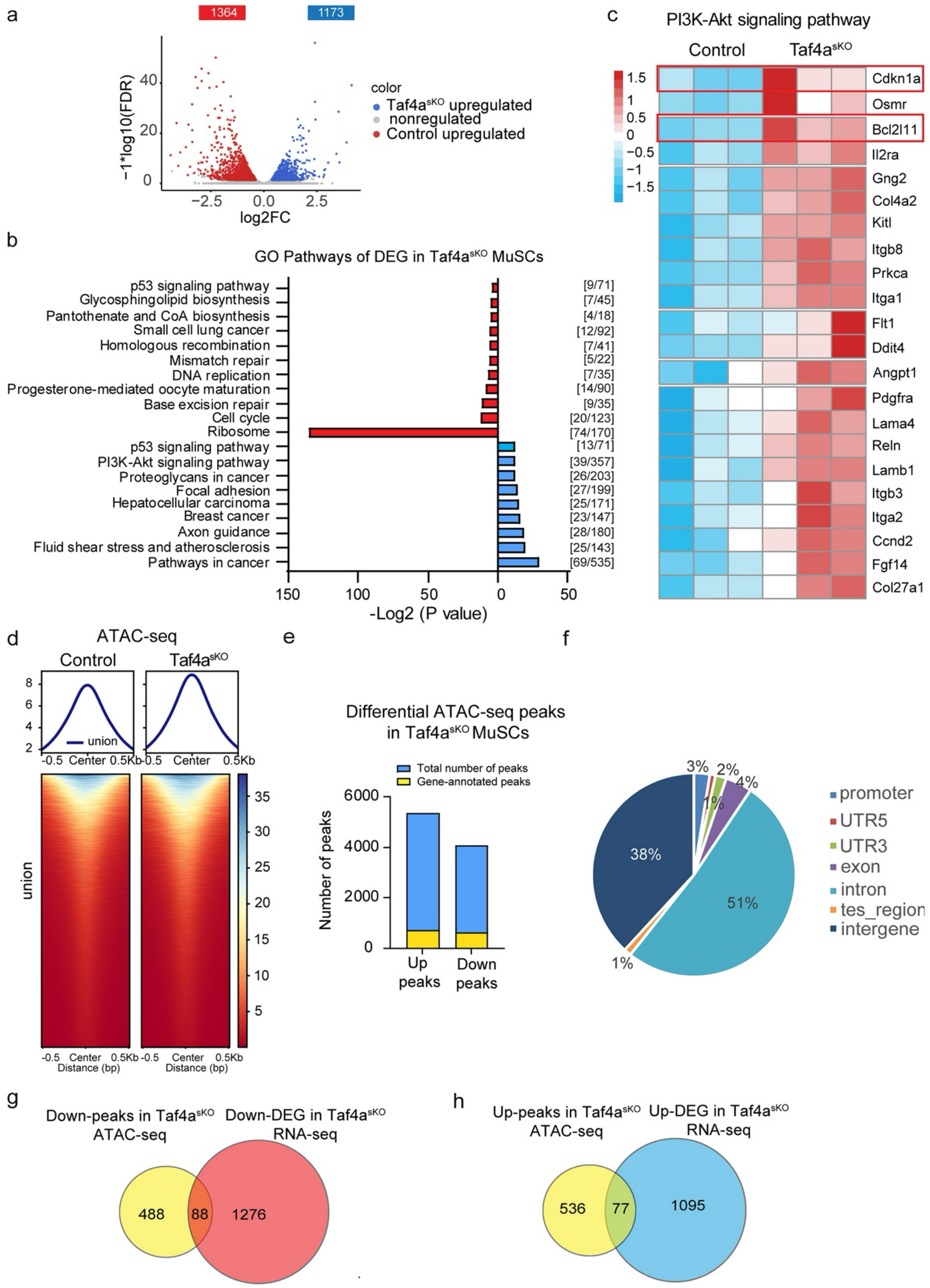

Supplementary Figure 3. Identification of genes and pathway affected by inactivation of *Taf4a* in MuSCs. a Volcano plot of differentially expressed genes in $Taf4a^{sKO}$ compared to control MuSCs. The vertical axis (y-axis) corresponds to the mean expression value of log10

(P value), and the horizontal axis (x-axis) displays the log2 fold change (FC) value. Red dots represent genes with decreased expression; blue dots represent genes with increased expression in *Taf4a*^{sKO} MuSCs. **b** Differentially expressed genes were categorized by GO terms based on P-values using EnrichR. **c** Heat map of genes with significantly increased expression in *Taf4a*^{sKO} MuSCs, involved in the PI3K/AKT signaling pathway (n=3). **d** Heat maps of centered ATAC-seq peaks in control and *Taf4a*^{sKO} MuSCs. The blue-to-red gradient indicates high-to-low counts in the corresponding region. **e** Number of differential ATAC-seq peaks in *Taf4a*^{sKO} MuSCs. **f** Genome-wide distribution of differential ATAC-seq peaks in *Taf4a*^{sKO} MuSCs. **g, h**Venn diagram of overlapping lost and gained ATAC-seq peaks with down- and up-DEG from RNA-seq data in control and *Taf4a*^{sKO} MuSCs.

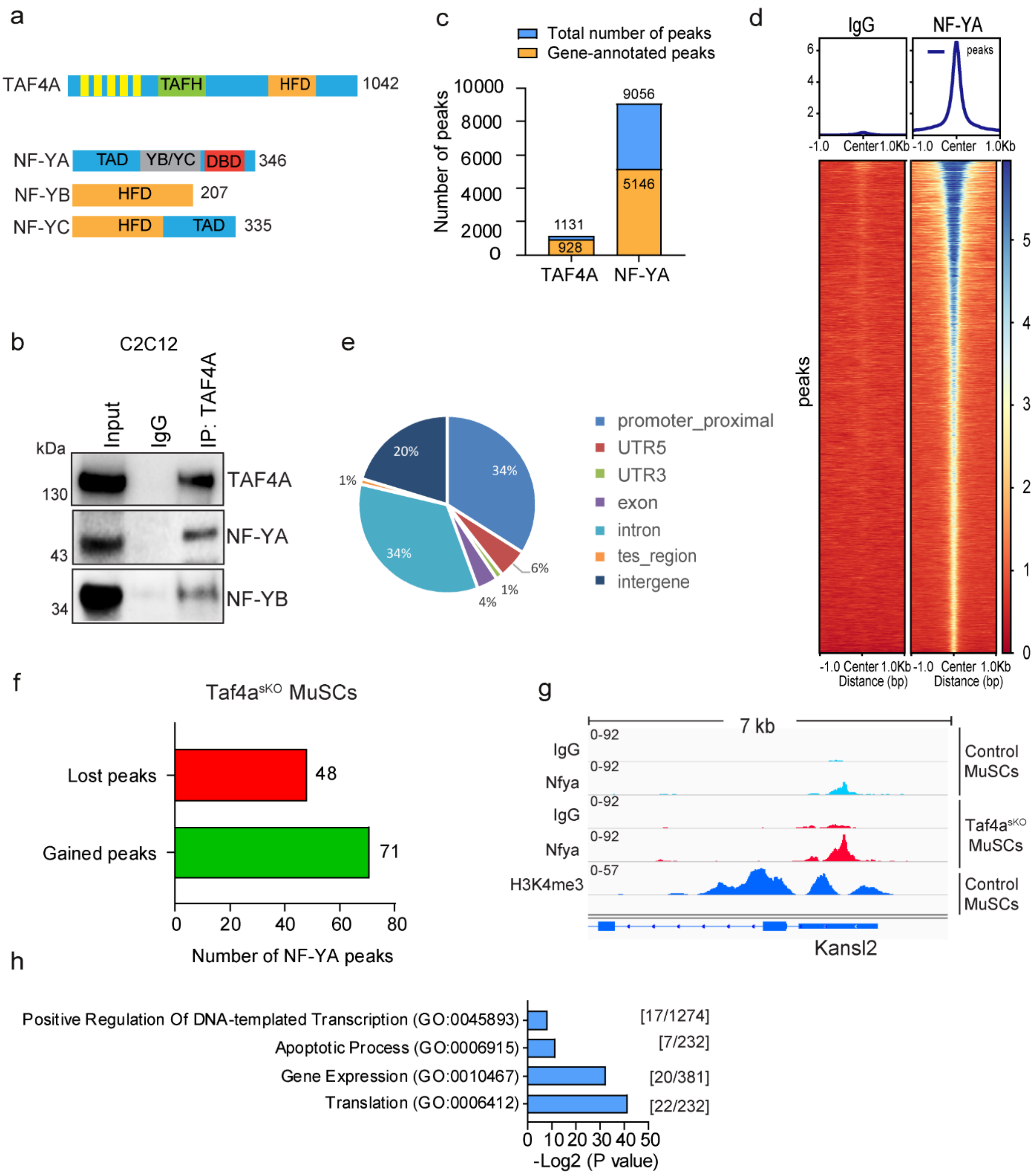

Supplementary Figure 4. DNA-binding of NF-YA is not affected by the absence of *Taf4a*. **a** Scheme of TAF4A and NF-Y TF protein domains. HFD – histone fold domain, TAF-H – TAF homology domain, TAD - transactivation domain, DBD - DNA binding domain, YB/YC – NF-YB/YC binding domain. **b** Co-IP of TAF4A and NF-YA/B in C2C12 cells. Blots were probed with antibodies against TAF4A and NF-YA or NF-YB (n=2). **c** Number of TAF4A and NF-YA CUT&RUN peaks in WT MuSCs. **d** Heat maps of centered NF-YA CUT&RUN peaks in wildtype MuSCs. The blue-to-red gradient indicates high-to-low counts in the corresponding region. **e** Genome-wide distribution of NF-YA peaks. **f** Number of lost and gained NF-YA peaks in *Taf4a*^{sKO} MuSCs compared to control MuSCs. **g** Distribution of NF-YA and H3K4me3

peaks in the proximal promoter region of the *Kansl2* gene in control and *Taf4a*^{sKO} MuSCs. **h** GO analysis of 159 genes with an overlap between TAF4A, NF-YA binding peaks and differential gene expression in *Taf4a*^{sKO} MuSCs. Genes were categorized based on P-values using EnrichR 6.8 (Fisher's exact test, two-sided).

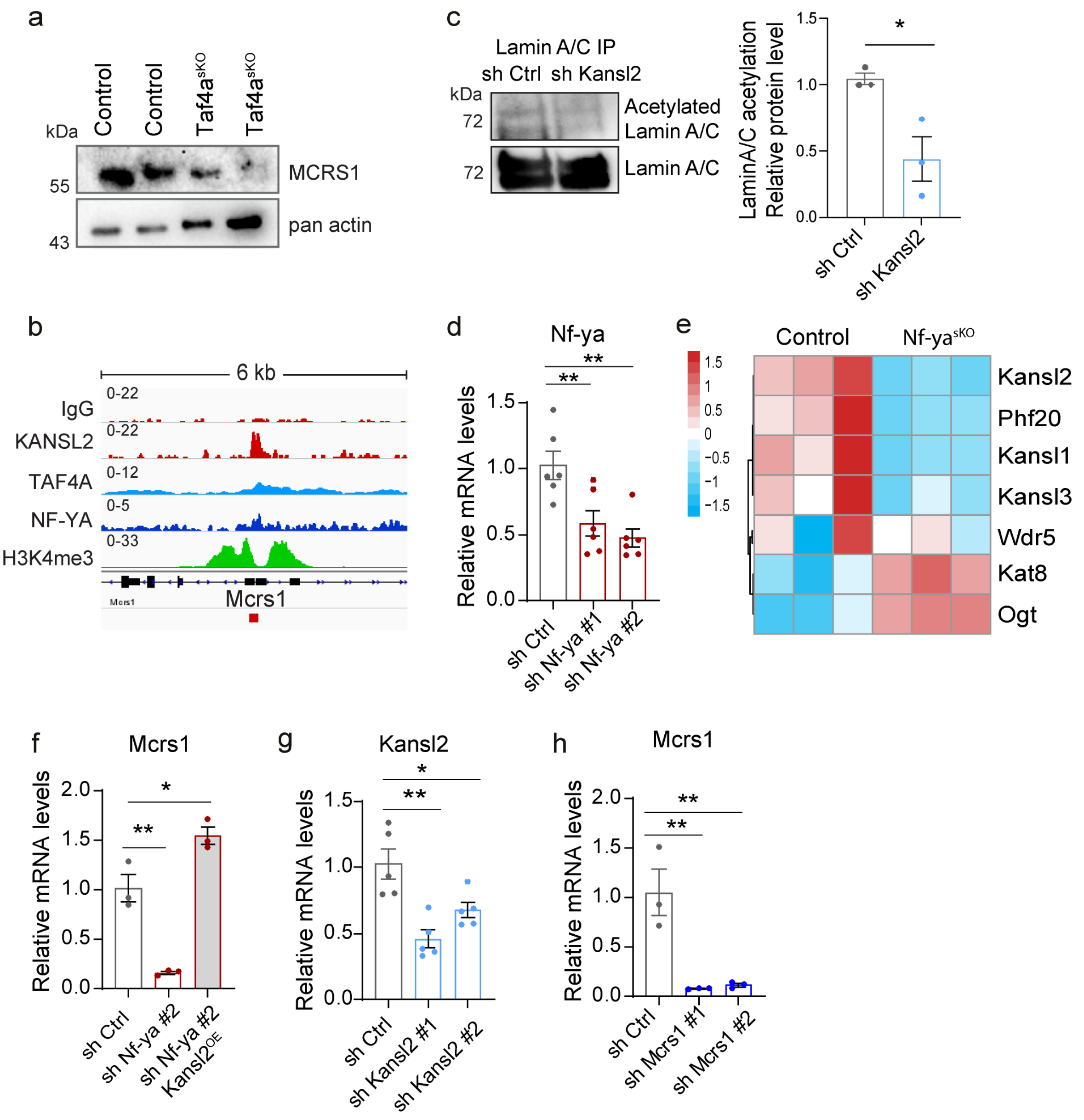

Supplementary Figure 5. *Kansl2* and *Mcrs1* are downregulated in *Nf-ya* knockdown MuSCs. a Western blot analysis of MCRS1 in MuSCs from control and *Taf4a*^{sKO}mice. Pan-actin was used as loading control (n=2). **b** KANSL2, TAF4A, NF-YA and H3K4me3 peaks in the proximal promoter region of the *Mcrs1* gene in WT MuSCs. **c** Western blot analysis of immunoprecipitated Lamin A/C to determine acetylation after knockdown of *Kansl2* in MuSCs. Acetylation signals were normalized to total Lamin A/C levels. Quantification of acetylation levels is shown on the right (unpaired two-tailed t-test: *p=0.0253, n=3). **d** RT-qPCR analysis of *Nf-ya* expression in scrambled control and *Nf-ya* knockdown MuSCs. *m36b4* was used as reference gene (one-way ANOVA with Bonferroni's multiple comparisons test: **p=0.0088, **p=0.0015, n=6). **e** Heat map of selected genes, including *Kansl2*, with reduced expression in *Nf-ya*-deficient MuSCs, based on the published dataset GSE154017 (n=3). **f** RT-qPCR analysis

of *Mcrs1* expression in scrambled control, *Nf-ya* knockdown, and *Nf-ya* knockdown *Kansl2* overexpressing MuSCs. *m36b4* was used as reference gene (one-way ANOVA with Bonferroni's multiple comparisons test: **p=0.0013, *p=0.0148, n=3). **g, h** RT-qPCR analysis of *Kansl2* (g) and *Mcrs1* (h) expression in scrambled control, *Kansl2* knockdown (g) and *Mcrs1* knockdown (h) MuSCs. *m36b4* was used as reference gene (one-way ANOVA with Bonferroni's multiple comparisons test: *p=0.0366, **p=0.0013 (g) n=5, **p=0.0048, **p=0.0059 (h) n=3). Data are presented as mean ± SEM. Source data are provided in the Source Data file.

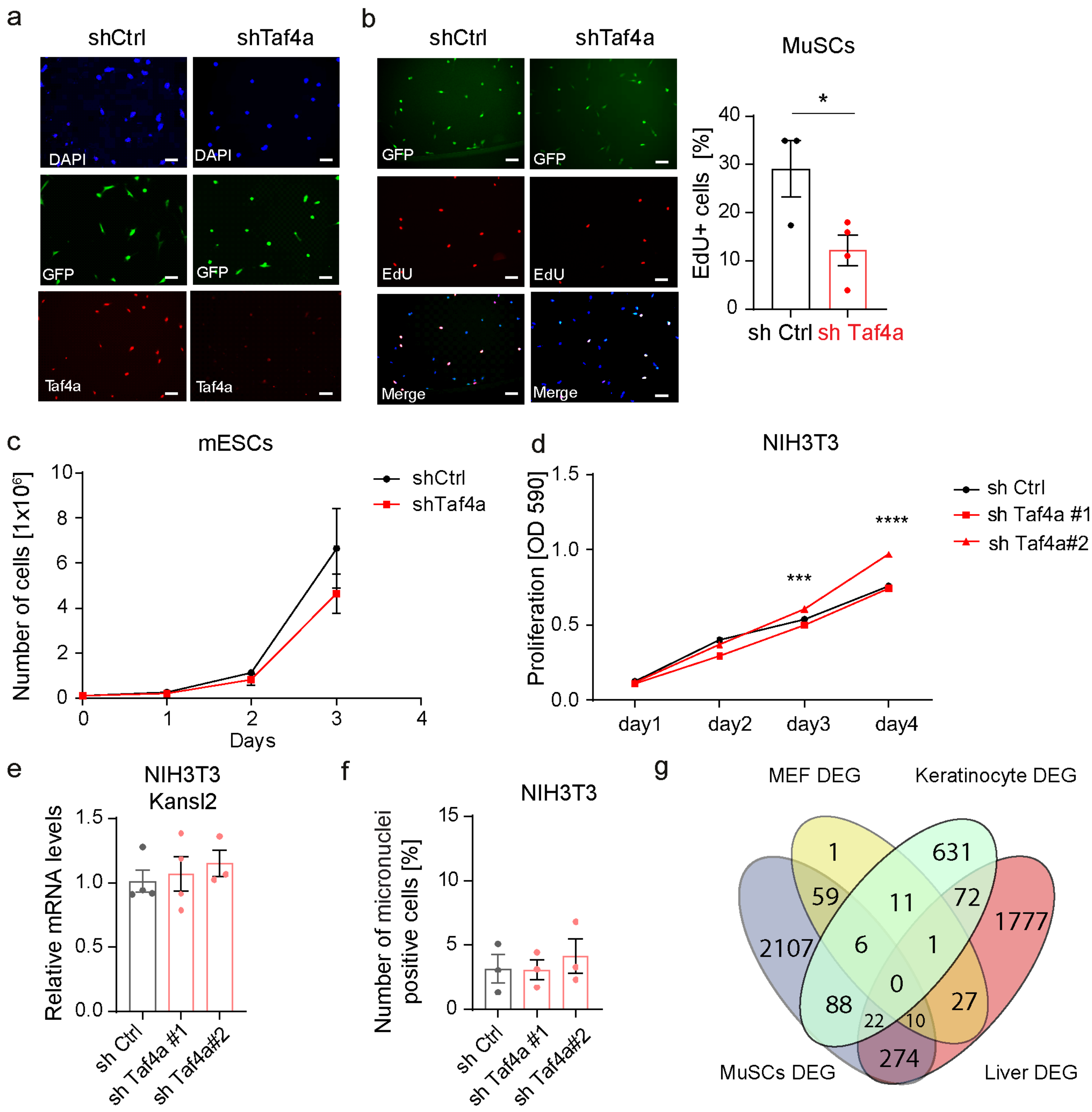

Supplementary Figure 6. Knockdown of *Taf4a* reduces proliferation of MuSCs but not of mESCs and NIH3T3 cells a Immunofluorescence staining of TAF4A in scrambled control and *Taf4a* knockdown MuSCs isolated from Pax7-zsgreen reporter mice by FACS-. Scale bar: 20 µm. **b** EdU incorporation and quantification of scrambled control (n=3) and *Taf4a* knockdown (n=4) MuSCs. Scale bar: 20 µm (unpaired two-tailed t-test: * p=0.0406). **c** Proliferation of mouse ESCs after knockdown of *Taf4a* (n=2). **d** Proliferation of mouse NIH3T3 cells after knockdown of *Taf4a* (two-way ANOVA with Bonferroni's multiple comparisons test: ***p=0,001 ****p<0.00001, n=4). **e** RT-qPCR analysis of *Kansl2* expression in scrambled control and *Taf4a* knockdown NIH3T3 cells. *m36b4* was used as reference gene (one-way

ANOVA with Bonferroni's multiple comparisons test: ns p>0.05 (n=4). **f** Number of micronuclei in scrambled control and *Taf4a* knockdown NIH3T3 cells (one-way ANOVA with Bonferroni's multiple comparisons test: ns p>0.05, n=3). **g** Venn diagram showing the overlap of differentially expressed genes (DEG), using RNA-seq data from freshly isolated $\textit{Taf4a}^{sKO}$ MuSCs, and published data from *Taf4a* knockout MEFs, keratinocytes and liver. Data are presented as mean ± SEM. Source data are provided in the Source Data file.

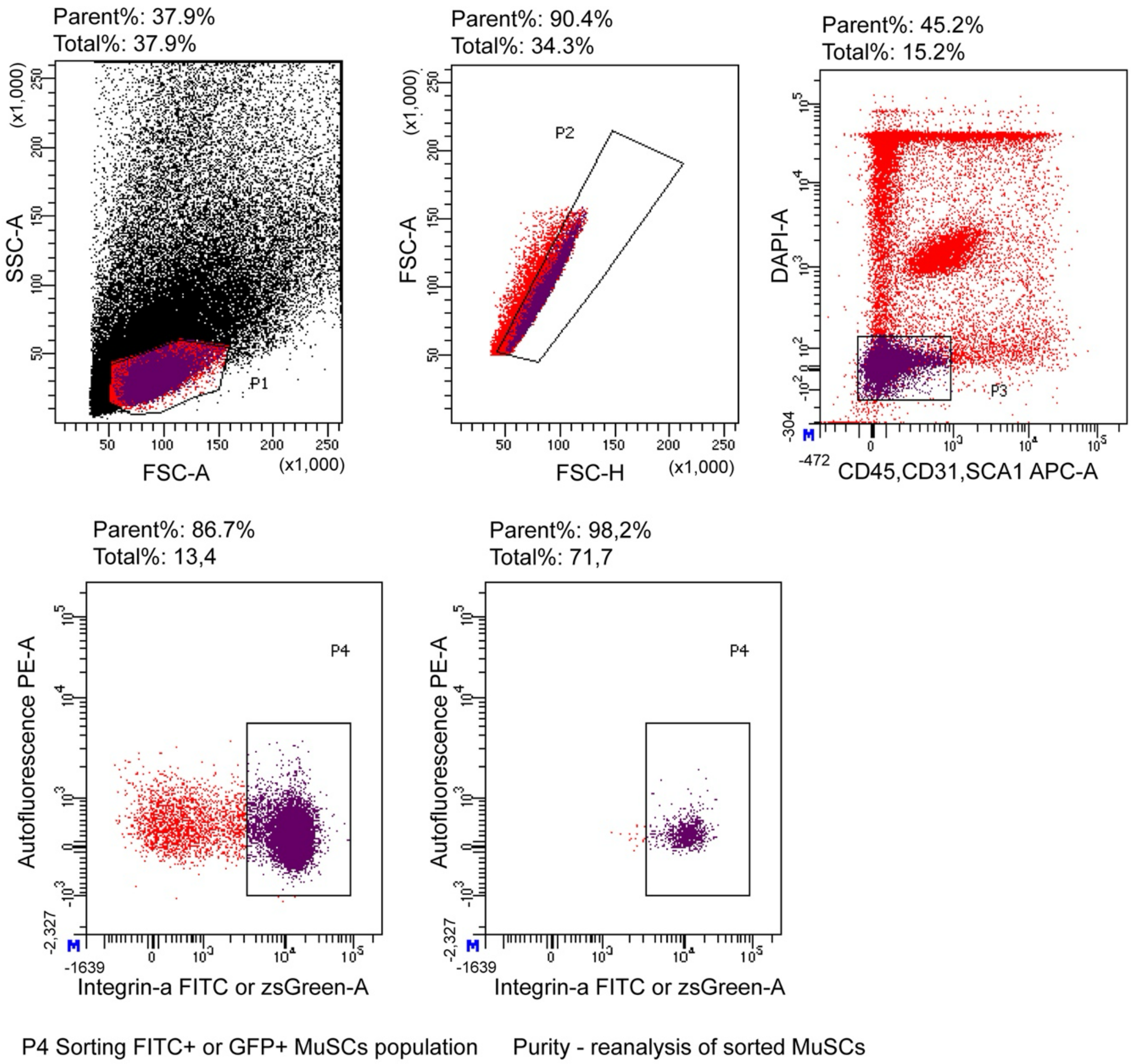

Supplementary Figure 7. Gating strategy for isolation of MuSCs. MuSCs were isolated by FACS based on negative selection for CD45, CD31, SCA1, APC-A and positive selection for FITC-integrin-α7 (upper panel) or by Pax7:-zsGreen, autofluorescence (lower left panel). MuSCs purity is shown in the lower right panel. Gates were defined using the CD FACS Diva v8 application. Parent and total percentages are shown in individual panels.

Supplementary Table 1. List of primers used for RT-qPCR and genotyping.

NAME	PRIMER SEQUENCE (5'-->3')	APPLICATION
**M36B4**	F-AAGGTGAAGCTGAAAGAACAG	RT-qPCR
	R-ATGGACGACAGGTGGGTACTG	RT-qPCR
**TAF4A**	F-GACGACGACAGATACGAGCA	RT-qPCR
	R-CTCTGGGTCTTCTTGTCTGGA	RT-qPCR
**KANSL2**	F-TAGTTACGTCTCGACGAAGAATG	RT-qPCR
	R-ATAGGGCCTGGGTTGCTC	RT-qPCR
**SMYD5**	F-GGCACCCCCTCAATAAGCTG	RT-qPCR
	R-ACCCAGTGGTCCTTGTCCTT	RT-qPCR
**SUV39H2**	F-CTGCCCAGGATAGCATTGTTC	RT-qPCR
	R-CAAGTCTCGGCTCCACATTTAC	RT-qPCR
**MCRS1**	F-GCAACCCGGAAGTAGATGTG	RT-qPCR
	R-TCCTCTCACAGTCCCAAAGC	RT-qPCR
**MYOD**	F-TAC AGT GGC GAC TCA GAT GC	RT-qPCR
	R-TAG TAG GCG GTG TCG TAG CC	RT-qPCR
**NF-YA**	F-GTTAATGGTGCAAGTCAGTGGA	RT-qPCR
	R-TCTGCTGTAAACCTTGTGTTCC	RT-qPCR
**TAF4A LOXP2**	F-GTGCTCCATGACTCTGGCAAG	Genotyping
	R-CTAGTTACTGCTCTGCACAAT	Genotyping
**PAX7CE**	F-ACTAGGCTCCACTCTGTCCTTC	Genotyping
	R-GCAGATGTAGGGACATTCCAGTG	Genotyping
**ZS GREEN**	F- CACAAAAACAGGTTAAACCCAG	Genotyping
	R- AGCACATAGGAGGCAGAGAC	Genotyping
**TOMATO**	F-CTCTGCTGCCTCCTGGCTTCT	Genotyping
	R-TCAATGGGCGGGGGTCGTT	Genotyping

RT-PCR: REAL-TIME QUANTITATIVE REVERSE TRANSCRIPTION PCR; F: FORWARD PRIMER; R: REVERSE PRIMER

Supplementary Table 2. List of antibodies used in the study and corresponding applications.

Antibody	Application (dilution)	Manufacturer	Cat. No.
**Anti-Pax7 mouse**	IF (1:1000)	R&D Systems	MAB1675
**Anti-MyoD rabbit**	IF (1:1000)	Santa Cruz	SC-304
**Anti-Gapdh**	WB (1:2000)	Cell signaling	(14C10)
**Actb**	WB (1:2000)	Sigma	A5441
**Rabbit IgG**	CnR (1:100)	Diagenode	C15410206
**Mouse IgG**	CnR (1:100)	Millipore	12-371B
**Anti-Sca1 APC**	FACS (1:100)	eBioscience	17-5981-83
**Anti-CD45 APC**	FACS (1:100)	eBioscience	17-0451-83
**Anti-CD31 APC**	FACS (1:100)	eBioscience	17-0311-82
**Anti-APC MicroBeads**	MACS	MACS	130-090-855
**Integrin-a7 FITC**	FACS (1:50)	MBL,JP	K0046-4
**H3**	WB (1:2000)	Cell Signaling	9715L
**Anti-γH2AX**	WB, IF (1:1000)	Cell Signaling	#2577
**Mcrs1**	WB (1:1000)	Proteintech	11362-1-AP
**Kansl2**	CnR (1:100)	Proteintech	27261-1-AP
**Taf4a (TAFIIp135(22))**	IF, CnR, WB (1:50), 6μg IP	Santa Cruz	Sc-136093
**NF-YA**	WB(1:1000), CnR (1:100)	Santa Cruz	Sc-17753
**Anti-V5**	WB (1:100)	Abcam	ab15828
**CalcR**	IF (1:100)	Abcam	ab11042
**GFP**	IF (1:1000)	Evrogen	AB513
**H3K9me3**	IF, WB (1:1000)	Abcam	ab8898
**H4K20me3**	WB (1:1000)	Abcam	ab9053
**H3K27me3**	WB (1:1000)	Millipore	07-449
**laminB1 (B-11)**	IF (1:1000)	Santa Cruz	Sc-374015
**Anti-P53 [PAb 240],**	WB (1:1000)	Abcam	ab26
**p-lamin A/C Ser 392**	WB (1:1000)	Aviva Systems Biology	OAAJ02346
**Lamin A/C (E-1)**	WB (1:1000), 6μg IP	Santa Cruz	Sc-376248
**Ac-lysine (AKL5C1)**	WB (1:1000)	Santa Cruz	Sc-32268
**NF-YB (G-2)**	WB (1:1000)	Santa Cruz	Sc-376546
**Pan-Actin**	WB (1:1000)	Cell signaling	4968
**Acetylated-Lysine**	WB (1:1000)	Cell signaling	9441
**Myod1**	IF (1:1000)	Abcam	ab133627
**mouse IgG / TrueBlot**	WB (1:1000)	eBioscience	18-8877-31
**rabbit IgG / TrueBlot**	WB (1:1000)	eBioscience	18-8816-31
**Anti-HA tag antibody**	WB (1:1000)	Abcam	ab9110
**Chicken anti-Goat IgG Alexa Fluor™ 594,**	IF (1:1000)	Invitrogen	A-21468
**Goat anti-Mouse IgG1 Alexa Fluor 488**	IF (1:1000)	Invitrogen	A-21121
**Goat anti-Rabbit IgG Alexa Fluor 594**	IF (1:1000)	Invitrogen	A-11012 A
**Goat anti-Rabbit IgG Alexa Fluor™ 680**	WB (1:1000)	Invitrogen	A-21076

IF: Immunofluorescence, WB: Western blot, CnR: Cut&Run, IP: Immunoprecipitation

Supplementary Table 3. List of shRNAs used in the study.

Name	**Clone ID**
shTaf4a #1	TRCN0000226289
shTaf4a #2	TRCN0000226290
shKansl2 #1	TRCN0000173179
shKansl2 #2	TRCN0000175794
shMcrs1 #1	TRCN0000175750
shMcrs1 #2	TRCN0000176378
shNfya #1	TRCN0000084439
shNfya #2	TRCN0000084441
shCtrl	TRCN0000087723
pMD2.G plasmid	Addgene # 12259
psPAX2 plasmid	Addgene # 12260

Supplementary Table 4. Identity of vectors for overexpression experiments.

Name	**Clone ID**
KANSL2	GenScript Biotech Clone ID:OMu51571C
