# Supplementary material for: Regulation of NSL by TAF4A is critical for genome stability and quiescence of muscle stem cells
Source: Nat Commun. 2025 Sep 30;16:8726. doi: 10.1038/s41467-025-64402-1 (PMC12484798; doi:10.1038/s41467-025-64402-1)
Supplement: Supplementary file 2 — Reporting Summary [file 41467_2025_64402_MOESM2_ESM.pdf]

nature portfolio

Corresponding author(s): Thomas Braun; Xuejun Yuan

Last updated by author(s): Aug 28, 2025

Reporting Summary

Nature Portfolio wishes to improve the reproducibility of the work that we publish. This form provides structure for consistency and transparency in reporting. For further information on Nature Portfolio policies, see our Editorial Policies and the Editorial Policy Checklist.

Statistics

For all statistical analyses, confirm that the following items are present in the figure legend, table legend, main text, or Methods section.

n/a	Confirmed	
☐	☒	The exact sample size (*n*) for each experimental group/condition, given as a discrete number and unit of measurement
☐	☒	A statement on whether measurements were taken from distinct samples or whether the same sample was measured repeatedly
☐	☒	The statistical test(s) used AND whether they are one- or two-sided<br>*Only common tests should be described solely by name; describe more complex techniques in the Methods section.*
☒	☐	A description of all covariates tested
☐	☒	A description of any assumptions or corrections, such as tests of normality and adjustment for multiple comparisons
☐	☒	A full description of the statistical parameters including central tendency (e.g. means) or other basic estimates (e.g. regression coefficient) AND variation (e.g. standard deviation) or associated estimates of uncertainty (e.g. confidence intervals)
☐	☒	For null hypothesis testing, the test statistic (e.g. *F*, *t*, *r*) with confidence intervals, effect sizes, degrees of freedom and *P* value noted<br>*Give P values as exact values whenever suitable.*
☒	☐	For Bayesian analysis, information on the choice of priors and Markov chain Monte Carlo settings
☒	☐	For hierarchical and complex designs, identification of the appropriate level for tests and full reporting of outcomes
☒	☐	Estimates of effect sizes (e.g. Cohen's *d*, Pearson's *r*), indicating how they were calculated

Our web collection on statistics for biologists contains articles on many of the points above.

Software and code

Policy information about availability of computer code

Data collection	Zeiss Z1.Imager - software Zen2 6.1.7601<br>Leica Sp8 - Software LAS X 3.5.7.23225<br>FACS AriaTM III (BD Biosciences) - BD FACS Diva v8 Software<br>LI-COR Odyssey<br>JPK NanoWizard 4XP (Bruker Nano) atomic force microscope mounted on a Zeiss Axio Observer<br>Philips CM10
Data analysis	GraphPad PRISM 10<br>StepOne Software v2.3<br>ImageJ 1.53<br>R 3.4<br>Trimmomatic version 0.39<br>STAR 2.6.0c<br>featureCounts 1.6.0<br>DESeq2 version 1.14.1<br>STAR 2.7.9a<br>Picard 2.25.5 (https://broadinstitute.github.io/picard/)<br>Trimmomatic version 0.39<br>Macs version 2.1.1 + 3.0.0a7

DESeq2 version 1.30.0
IGV version 2.4.10
JPK SPM Control Software v 7.0.165
DAVID Bioinformatics Resources 6.8
EnrichR
Fiji v1.54f

RNA-seq analysis
Trimmomatic version 0.39 was employed to trim reads after a quality drop below a mean of Q15 in a window of 5 nucleotides and keeping only filtered reads longer than 15 nucleotides (Bolger et al., Trimmomatic: a flexible trimmer for Illumina sequence data).
Reads were aligned versus Ensembl mouse genome version mm10 (Ensembl release 101) with STAR 2.6.0c (Dobin et al., STAR: ultrafast universal RNA-seq aligner).
Alignments were filtered to remove multi-mapping reads.
Gene counts were established with featureCounts 1.6.0 by aggregating reads overlapping exons excluding those overlapping multiple genes (Liao et al., featureCounts: an efficient general purpose program for assigning sequence reads to genomic features).
The raw count matrix was normalized with DESeq2 version 1.14.1 (Love et al., Moderated estimation of fold change and dispersion for RNA-Seq data with DESeq2).

CUT&RUN
DNA was quantified by Qubit and max. 10ng DNA was used as input for SMARTer® ThruPLEX® DNA-seq Kit following manufacture's protocol (Takara Bio). Sequencing was performed either on NextSeq500 platform (Illumina) using P3 flowcell with 2x38bp single-end setup or on NextSeq2000 platform (Illumina) using P3 flowcell with 2x61bp single-end setup or 2x 36bp. Trimmomatic version 0.39 was employed to trim reads after a quality drop below a mean of Q15 in a window of 5 nucleotides and keeping only filtered reads longer than 15 nucleotides (Bolger et al., Trimmomatic: a flexible trimmer for Illumina sequence data).
Reads were aligned versus Ensembl mouse genome version mm10 (Ensembl release 101) with STAR 2.7.9a (Dobin et al., STAR: ultrafast universal RNA-seq aligner).
Alignments were filtered to remove: duplicates with Picard 2.25.5 (Picard: A set of tools (in Java) for working with next generation sequencing data in the BAM format), spliced, multi-mapping, ribosomal, or mitochondrial reads.
Peak calling was performed with Macs version 2.1.1 with FDR < 0.05 and additional parameters "--extsize 100 --nomodel --to-large" (Zhang et al., Model-based Analysis of ChIP-Seq) (GSE277867,GSE277868,GSE277869) and Macs version 3.0.0a7 with FDR < 0.1 and additional parameters "--extsize 75 --nomodel --to-large" (GSE277870).
Remaining peaks were unified to represent a common set of regions for all samples.
Counts were produced with featureCounts (Liao et al., featureCounts: an efficient general purpose program for assigning sequence reads to genomic features).
The raw count matrix was normalized with DESeq2 version 1.30.0 (Love et al., Moderated estimation of fold change and dispersion for RNA-Seq data with DESeq2).
Peaks were annotated with the promoter (TSS +- 10000 nt) of the nearest gene based on Ensembl data.

ATAC-seq
Libraries were mixed in equimolar ratios and sequenced by the NextSeq500 platform using V2 chemistry with paired-end mode. Trimmomatic version 0.39 was employed to trim reads after a quality drop below a mean of Q15 in a window of 5 nucleotides and keeping only filtered reads longer than 15 nucleotides (Bolger et al., Trimmomatic: a flexible trimmer for Illumina sequence data). Reads were aligned versus Ensembl mouse genome version mm10 (Ensembl release 101) with STAR 2.7.11b (Dobin et al., STAR: ultrafast universal RNA-seq aligner). Alignments were filtered to remove: duplicates with Picard 3.1.1 (Picard: A set of tools (in Java) for working with next generation sequencing data in the BAM format), spliced, multi-mapping, ribosomal, or mitochondrial reads. Peak calling was performed with Macs version 3.0.0a7 with FDR < 0.0001 (Zhang et al., Model-based Analysis of ChIP-Seq). Remaining peaks were unified to represent a common set of regions for all samples. Counts were produced with featureCounts (Liao et al., featureCounts: an efficient general purpose program for assigning sequence reads to genomic features). The raw count matrix was normalized for sequencing depth. Peaks were annotated with the promoter (TSS +- 10000 nt) of the nearest gene based on Ensembl release 101. Contrasts were created with DESeq2 based on the normalized count peak matrix with all size factors set to one. Peaks were classified as significantly differential at average count > 10 and -1 < log2FC > 1.

For manuscripts utilizing custom algorithms or software that are central to the research but not yet described in published literature, software must be made available to editors and reviewers. We strongly encourage code deposition in a community repository (e.g. GitHub). See the Nature Portfolio guidelines for submitting code & software for further information.

Data

Policy information about availability of data

All manuscripts must include a data availability statement. This statement should provide the following information, where applicable:

- Accession codes, unique identifiers, or web links for publicly available datasets
- A description of any restrictions on data availability
- For clinical datasets or third party data, please ensure that the statement adheres to our policy

Raw and processed CUT&RUN data for TAF4A, NF-YA and KANSL2 in wild type MuSCs, ATAC-seq data, NF-YA CUT&RUN data in control and Taf4asKO MuSCs, and RNA-seq data of freshly isolated WT and Taf4asKO MuSCs are available in the NCBI Gene Expression Omnibus (GEO), under accession number GSE277872 [https://www.ncbi.nlm.nih.gov/geo/query/acc.cgi?acc=GSE277872]. Published H3K4me3 ChIP-seq data were downloaded from GEO time-point T3 from "GSE103163 [https://www.ncbi.nlm.nih.gov/geo/query/acc.cgi?acc=GSE103163]": GSM2756400 and GSM2756401. Published RNA-seq data of freshly isolated and activated/proliferating WT MuSCs were downloaded from GEO under accession number "GSE108040" [https://www.ncbi.nlm.nih.gov/geo:%20GSE108040]. Published RNA-seq data of freshly isolated WT MuSCs and WT muscle were downloaded from GEO under accession number "GSE199487" [https://www.ncbi.nlm.nih.gov/geo/query/acc.cgi?acc=GSE199487] and GSE168984, [https://www.ncbi.nlm.nih.gov/geo/query/acc.cgi?acc=GSE168984], respectively. Published RNA-seq data of WT

and NF-YA knockout MuSCs were downloaded from GEO under accession number "GSE154017" [https://www.ncbi.nlm.nih.gov/geo/query/acc.cgi?acc=GSE154017].

GEO accession number: GSE277872 Fig. 1g, Fig. 2a, 2b,2c Fig. 3a-d, 3g-i , Fig 4b, 4f-j, Supp. Fig. 3 a-h, Supp. Fig. 4a, 4c-h and Supp. Fig. 5b are associated with this data.
publicly available datasets:
GSE103163 (GSM2756400 and GSM2756401) - Fig. 3i and Supp. fig. 4g and Supp. Fig. 5b
GSE108040 - Fig. 2a
GSE199487 and GSE168984 - Supp. Fig. 1b
GSE154017 - Supp. Fig. 5e

Research involving human participants, their data, or biological material

Policy information about studies with human participants or human data. See also policy information about sex, gender (identity/presentation), and sexual orientation and race, ethnicity and racism.

Reporting on sex and gender	Not applicable
Reporting on race, ethnicity, or other socially relevant groupings	Not applicableNot applicable
Population characteristics	Not applicable
Recruitment	Not applicable
Ethics oversight	Not applicable

Note that full information on the approval of the study protocol must also be provided in the manuscript.

Field-specific reporting

Please select the one below that is the best fit for your research. If you are not sure, read the appropriate sections before making your selection.

[x] Life sciences [ ] Behavioural & social sciences [ ] Ecological, evolutionary & environmental sciences

For a reference copy of the document with all sections, see nature.com/documents/nr-reporting-summary-flat.pdf

Life sciences study design

All studies must disclose on these points even when the disclosure is negative.

Sample size	Sample size were determined based on established practice and applicable standards. We opted for sample sizes which are commonly used sample sizes in the field.<br>For in vivo studies, a minimum of three biological replicates was analyzed. Each experiment in which data were quantified was performed with at least 3 replicates.
Data exclusions	No data were excluded.
Replication	All in vivo studies were performed once with indicated numbers of animals. Sample sizes and statistical analyses and significance levels are all indicated in the figure legends or the method part.
Randomization	All animals were numbered and experiments were performed in a blinded pattern. After data collection, genotypes were revealed and animals assigned to different groups for analysis.
Blinding	In vivo experiments were performed in a blinded pattern.<br>In vitro experiments were not blinded during data collection or analysis. Positive controls, negative controls and target samples were analyzed in exactly the same manner.

Reporting for specific materials, systems and methods

We require information from authors about some types of materials, experimental systems and methods used in many studies. Here, indicate whether each material, system or method listed is relevant to your study. If you are not sure if a list item applies to your research, read the appropriate section before selecting a response.

Materials & experimental systems

n/a	Involved in the study	
☐	☒	Antibodies
☐	☒	Eukaryotic cell lines
☒	☐	Palaeontology and archaeology
☐	☒	Animals and other organisms
☒	☐	Clinical data
☒	☐	Dual use research of concern
☒	☐	Plants

Methods

n/a	Involved in the study	
☐	☒	ChIP-seq
☐	☒	Flow cytometry
☒	☐	MRI-based neuroimaging

Antibodies

Antibodies used

Anti-Pax7 mouse, R&D Systems MAB1675, IF (1:1000)
Anti-MyoD rabbit, Santa Cruz SC-304, IF (1:1000)
Anti-Gapdh, Cell signaling (14C10), WB (1:2000)
Actb, Sigma A5441, WB (1:2000)
Rabbit IgG, Diagenode C15410206, CnR (1:100)
Mouse IgG, Millipore 12-371B, CnR (1:100)
Anti-Sca1 APC, eBioscience 17-5981-83, FACS (1:100)
Anti-CD45 APC, eBioscience 17-0451-83, FACS (1:100)
Anti-CD31 APC, eBioscience 17-0311-82, FACS (1:100)
Anti-APC MicroBeads MACS MACS 130-090-855
Integrin-a7 FITC, MBL,JP K0046-4, FACS (1:50)
Hisone H3, Cell Signaling 9715L, WB (1:2000)
Anti-γH2AX, Cell Signaling #2577, WB, IF (1:1000)
Mcrs1, Proteintech 11362-1-AP, WB (1:1000)
Kansl2, Proteintech 27261-1-AP, CnR (1:100)
Taf4a (TAFIIp135(22)), Santa Cruz Sc-136093, IF, CnR, WB (1:50)
NF-YA, Santa Cruz Sc-17753, CnR (1:100), WB(1:1000)
NF-YB (G-2), Santa Cruz Sc-376546 WB(1:1000)
Anti-V5, Abcam ab15828, WB (1:100)
CalcR, Abcam ab11042, IF (1:100)
GFP, Evrogen AB513, F (1:1000)
H3K9me3, Abcam ab8898, IF, WB (1:1000)
H4K20me3, Abcam ab9053, WB (1:1000)
H3K27me3, Millipore 07-449, WB (1:1000)
Lamin B1 (S-20), Santa Cruz sc-30264, IF (1:1000)
Anti-P53 [PAb 240], Abcam, ab26 WB (1:1000)
p-Lamin A/C Ser 392, Aviva Systems Biology OAAJ02346, WB (1:1000)
mouse IgG / TrueBlot, Rockland 18-8817-33, WB (1:1000)
rabbit IgG / TrueBlot, Rockland 18-8816-33, WB (1:1000)
Anti-HA tag antibody, Abcam ab9110, WB (1:1000)
Chicken anti-Goat IgG Alexa Fluor™ 594, Invitrogen # A-21468, IF (1:1000)
Goat anti-Mouse IgG1 Alexa Fluor 488, Invitrogen # A-21121 IF (1:1000)
Goat anti-Rabbit IgG Alexa Fluor 594, Invitrogen # A-11012 A-IF (1:1000)
Lamin A/C (E-1) WB (1:1000), 6μg IP Santa Cruz Sc-376248
Ac-lysine (AKL5C1) WB (1:1000) Santa Cruz Sc-32268
NF-YB WB (1:1000) Santa Cruz Sc-376546
Acetylated-Lysine WB (1:1000) Cell signaling 9441
Myod1 IF (1:1000) Abcam ab133627

Validation

Pax7 (1:1000 R&D Systems MAB1675) validated by immunocytochemistry in C2C12 Mouse Cell Line; MyoD (1:1000 Santa Cruz SC-304) validated by western blot analysis in HeLa (A), HL-60 (B), SJRH30 (C), NIH/3T3 (D) and RD (E) whole cell lysates and A-673 nuclear extract (F); Gapdh (WB 1:2000 Cell signaling (14C10) validated by western blot analysis of extracts from various cell lines, immunofluorescent analysis of HeLa cells; Actb, (Sigma A5441, WB (1:2000) validated by knockdown; IgG (ChIP Diagenode C15410206) validated by ChIP assays and immunofluorescence in HeLA cells; Mouse IgG (Millipore 12-371) Normal Mouse IgG Polyclonal Antibody control validated for use in Immunoprecipitation & Western Blotting.; Sca1 -APC (FACS 1:100, eBioscience 17-5981-83) validated by FACS analysis of stained of unstimulated and 4-day Con A-stimulated BALB/c splenocytes; CD45-APC (FACS 1:100, eBioscience 17-0451-83) validated by FACS analysis in C57BL/6 mouse bone marrow cells; CD31 APC (FACS 1:100, eBioscience 17-0311-82) validated by FACS analysis in C57BL/6 bone marrow cells; Integrin-a7 FITC (FACS 1:100, MBL,JP K0046-4);H3 (WB 1:2000, ChIP 2Cell Signaling 9715L)validated by western blot analysis of extracts from various cell lines; Anti-γH2AX, (Cell Signaling #2577) was validated by western blot analysis of extracts from 293 cells, untreated or UV-treated. Mcrs1, Proteintech 11362-1-AP, WB (1:1000) was validated by western blot analysis of extracts from mouse uterus tissue, mouse spleen tissue, human spleen tissue, human liver tissue, human brain tissue, Jurkat and HeLa cells; Kansl2 (Proteintech 27261-1-AP) was validated by western blot analysis of mouse heart tissue; Taf4a (TAFIIp135(22)), Santa Cruz Sc-136093 - was validated by western blot analysis of TAF II p135 expression in HeLa

nuclear extract, ES-D3 whole cell lysate, Jurkat whole cell lysate and mouse ovary tissue extract. Immunofluorescence staining of HeLa cells showing nuclear staining; NF-YA (Santa Cruz Sc-17753) was validated by western blot analysis of NF-YA expression in NIH/3T3 whole cell lysate, western blot analysis of human recombinant NF-YA fusion protein. Immunofluorescence staining of formalin-fixed Hep G2 cells showing nuclear localization; NF-YB (G-2), Santa Cruz Sc-376546 was validated by western blot analysis of in RAW 264.7 nuclear extract and HeLa, A549, NIH/3T3, A-10, HEK293 and C6 whole cell lysates. Immunofluorescence staining of methanol-fixed HeLa cells. ICC of human small intestine tissue, human kidney tissue, human seminal vesicle tissue and human prostate tissue; Anti-V5 (Abcam ab15828) was validated by western blot of Recombinant V5 tagged Groucho homolog and Recombinant V5 tagged Groucho and ChIP using chromatin prepared from a stably transfected 293T human cell line harbouring the GAL4 upstream activation sequence that a transiently transfected with a V5 or T7- tagged GAL4 DNA Binding Domain construct; CalcR, Abcam ab11042; GFP (Evrogen AB513) was validated by western blot of lisates of HEK293 cells expressing different fluorescent proteins; H3K9me3(Abcam ab8898) was validated by western blot of Calf Thymus Histone Preparation Nuclear Lysate and the signal is blocked by the addition of the immunizing peptide; H4K20me3 (Abcam ab9053) was validated by western blot of Calf Thymus Histone Preparation Nuclear Lysate and the signal is blocked by the addition of the immunizing peptide; H3K27me3 (Millipore 07-449) was validated by dotblot using histone peptides with various modifications; Lamin B1 (S-20)(Santa Cruz sc-30264); Anti-P53 [PAb 240] (Abcam, ab26) was validated by western blot in wild type HCT116 cells treated with irinotecan and p53 knockout HCT116 cells; p-Lamin A/C Ser 392 (Aviva Systems Biology OAAJ02346) was validated by western blot analysis in HeLa whole cell lysates and treated with the antigen-specific peptide; mouse IgG / TrueBlot (Rockland 18-8817-33) was validated by western blot in IPs in various human and mouse cell lines in comparison of using conventional HRP-conjugated anti-mouse polyclonal antibodies; rabbit IgG / TrueBlot (Rockland 18-8816-33) was validated by western blot in IP in various human and mouse cell lines; Anti-HA tag antibody ChIP Abcam ab9110 validated by western blot with a nuclear lysate of HEK293T cells transiently expressing HA-tagged protein; Chicken anti-Goat IgG Alexa Fluor™ 594 (Invitrogen # A-21468) - the specificity of the secondary antibody was proved by the absence of signal in HeLa (negative model) due to no primary antibody binding; Goat anti-Mouse IgG1 Alexa Fluor 488 (Invitrogen # A-21121)- the specificity of the secondary antibody was proved by the absence of signal in HeLa (negative model) due to no primary antibody binding; Goat anti-Rabbit IgG Alexa Fluor 594 (Invitrogen # A-11012) - the specificity of the secondary antibody was proved by the absence of signal in HeLa (negative model) due to no primary antibody binding;Lamin A/C (E-1) was validated by direct fluorescent western blot analysis of Lamin A/C expression in C32, PC-3, HeLa, A-431, NIH/3T3 and Sol8 whole cell lysates;Ac-lysine (AKL5C1) was validated by western blot analysis of Ac-lysine acetylation in untreated and Trichostatin A (sc-3511) treated NIH/3T3 whole cell lysates; NF-YB (G-2) was validated western blot analysis of NF-YB expression in RAW 264.7 nuclear extract (A) and HeLa (B), A549 (C), NIH/3T3 (D), A-10 (E) and C6 (F) whole cell lysates.; Acetylated-Lysine Antibody was validated by western blot analysis from COS cells, untreated or TSA-treated. Specificity and sensitivity of acetylated-lysine antibody was assayed on acetylated BSA or nonacetylated BSA; Anti-MyoD1 antibody was validated in RD (Human muscle rhabdomyosarcoma) whole cell lysate, as negative controls were used whole cell lysate of HEK-293, HeLa cells.

Eukaryotic cell lines

Policy information about cell lines and Sex and Gender in Research

Cell line source(s)

v6.5 mESCs NBP1-41162
C2C12 ATCC #CRL-1772
NIH3T3 ATCC CRL-1658

Authentication

Each cell line used was morphologically confirmed according to the information provided by the culture collections.

Mycoplasma contamination

Tested for being Mycoplasma free

Commonly misidentified lines
(See ICLAC register)

No commonly misidentified cell lines were used

Animals and other research organisms

Policy information about studies involving animals; ARRIVE guidelines recommended for reporting animal research, and Sex and Gender in Research

Laboratory animals

The following mouse strains were used in the study:

Wild type mice C57BL/6
Pax7:ZsGreen reporter mice
Control: Pax7CE+/+ // Taf4afl/fl// Pax7 Zs green neg/pos or Pax7CE+/p // Taf4afl/fl// Zs green neg/pos.
Taf4asKO : Pax7CE+/p // Taf4afl/fl// Pax7 Zs green neg/pos with tamoxifen treatment

All mice were maintained on a C57BL/6 background and littermates were used as controls in all experiments.

All mice used in this study were drug and test naive, healthy prior to the studies, not used in previous procedures and maintained in individually ventilated cages, at 22.5°C ± 1 °C and a relative humidity of 50% ± 5% with controlled illumination {12 h dark/light cycle). Mice were given ad libitum access to food and water. All mouse strains were backcrossed and maintained on a C57BL/6 genetic background. Female and male animals at the age between 8 to 20 weeks to equal proportions were analyzed in this study. None of the determined parameters in this study correlated with animal sex.

Wild animals

Studies did not involve wild animals.

Reporting on sex

Female and male adult animals to equal proportions were analyzed in this study. None of the determined parameters in this study correlated with animal sex.

Field-collected samples	Studies did not involve samples collected in the field.
Ethics oversight	All animal experiments were done in accordance with the Guide for the Care and Use of Laboratory Animals published by the US National Institutes of Health (NIH Publication No. 85-23, revised 1996) and were approved by the responsible Committee for Animal Rights Protection of the State of Hessen (Regierungspraesidium Darmstadt, Wilhelminenstr. 1-3, 64283 Darmstadt, Germany) with the project number B2/1137 and B2/2048.

Note that full information on the approval of the study protocol must also be provided in the manuscript.

Plants

Seed stocks	Not applicable
Novel plant genotypes	Not applicable
Authentication	Not applicable

ChIP-seq

Data deposition

☒ Confirm that both raw and final processed data have been deposited in a public database such as GEO.

☒ Confirm that you have deposited or provided access to graph files (e.g. BED files) for the called peaks.

Data access links
May remain private before publication.

https://www.ncbi.nlm.nih.gov/geo/query/acc.cgi?acc=GSE277872

Files in database submission

The *_processed.txt files contain unified peaks per dataset that can be extracted as BED.
MuSC_WT_Taf4a_1_R1.fastq.gz
MuSC_WT_Taf4a_1_igg_R1.fastq.gz
MuSC_WT_Taf4a_2_R1.fastq.gz
MuSC_WT_Taf4a_2_igg_R1.fastq.gz
MuSC_WT_Taf4a_3_R1.fastq.gz
MuSC_WT_Taf4a_3_igg_R1.fastq.gz
MuSC_WT_Taf4a_1_R2.fastq.gz
MuSC_WT_Taf4a_1_igg_R2.fastq.gz
MuSC_WT_Taf4a_2_R2.fastq.gz
MuSC_WT_Taf4a_2_igg_R2.fastq.gz
MuSC_WT_Taf4a_3_R2.fastq.gz
MuSC_WT_Taf4a_3_igg_R2.fastq.gz
MuSC_WT_Nfya_1_R1.fastq.gz
MuSC_WT_Nfya_1_igg_R1.fastq.gz
MuSC_WT_Nfya_2_R1.fastq.gz
MuSC_WT_Nfya_2_igg_R1.fastq.gz
MuSC_WT_Nfya_3_R1.fastq.gz
MuSC_WT_Nfya_3_igg_R1.fastq.gz
MuSC_WT_Nfya_1_R2.fastq.gz
MuSC_WT_Nfya_1_igg_R2.fastq.gz
MuSC_WT_Nfya_2_R2.fastq.gz
MuSC_WT_Nfya_2_igg_R2.fastq.gz
MuSC_WT_Nfya_3_R2.fastq.gz
MuSC_WT_Nfya_3_igg_R2.fastq.gz
MuSC_WT_Kansl2_1_R1.fastq.gz
MuSC_WT_Kansl2_1_igg_R1.fastq.gz
MuSC_WT_Kansl2_2_R1.fastq.gz
MuSC_WT_Kansl2_2_igg_R1.fastq.gz
MuSC_WT_Kansl2_3_R1.fastq.gz
MuSC_WT_Kansl2_3_igg_R1.fastq.gz
MuSC_WT_Kansl2_4_R1.fastq.gz
MuSC_WT_Kansl2_4_igg_R1.fastq.gz
MuSC_WT_Kansl2_1_R2.fastq.gz
MuSC_WT_Kansl2_1_igg_R2.fastq.gz
MuSC_WT_Kansl2_2_R2.fastq.gz

MuSC_WT_Kansl2_2_igg_R2.fastq.gz
MuSC_WT_Kansl2_3_R2.fastq.gz
MuSC_WT_Kansl2_3_igg_R2.fastq.gz
MuSC_WT_Kansl2_4_R2.fastq.gz
MuSC_WT_Kansl2_4_igg_R2.fastq.gz
MuSC_WT_Nfya_1_R1.fastq.gz
MuSC_WT_Nfya_1_igg_R1.fastq.gz
MuSC_WT_Nfya_2_R1.fastq.gz
MuSC_WT_Nfya_2_igg_R1.fastq.gz
MuSC_Taf4a-KO_Nfya_1_R1.fastq.gz
MuSC_Taf4a-KO_Nfya_1_igg_R1.fastq.gz
MuSC_Taf4a-KO_Nfya_2_R1.fastq.gz
MuSC_Taf4a-KO_Nfya_2_igg_R1.fastq.gz
MuSC_WT_Nfya_1_R2.fastq.gz
MuSC_WT_Nfya_1_igg_R2.fastq.gz
MuSC_WT_Nfya_2_R2.fastq.gz
MuSC_WT_Nfya_2_igg_R2.fastq.gz
MuSC_Taf4a-KO_Nfya_1_R2.fastq.gz
MuSC_Taf4a-KO_Nfya_1_igg_R2.fastq.gz
MuSC_Taf4a-KO_Nfya_2_R2.fastq.gz
MuSC_Taf4a-KO_Nfya_2_igg_R2.fastq.gz
WT_1_R1.fastq.gz
WT_2_R1.fastq.gz
WT_3_R1.fastq.gz
ko-Taf4_1_R1.fastq.gz
ko-Taf4_2_R1.fastq.gz
ko-Taf4_3_R1.fastq.gz
WT_1_R2.fastq.gz
WT_2_R2.fastq.gz
WT_3_R2.fastq.gz
ko-Taf4_1_R2.fastq.gz
ko-Taf4_2_R2.fastq.gz
ko-Taf4_3_R2.fastq.gz
cnr_yua49_54_57_taf4a_processed.txt
cnr_yua54_63_nfya_processed.txt
cnr_yua57_63_kansl2_processed.txt
cnr_yua113_nfya_processed.txt
atac_yua113_atac_2_processed.txt

Genome browser session
(e.g. UCSC)

N/A

Methodology

Replicates

Three biological replicates of ATAC-seq, TAF4A and NF-YA, four of KANSL2 CUT&RUN in wild type MuSCs and two replicates of NF-YA in control wild type and Taf4asKO MuSCs.

Sequencing depth

Sample, total reads, uniquely mapped reads, read length, single/paired
GSM8533638 MuSC_WT_Taf4a_1, 34870486, 26457935, 38, paired-end
GSM8533639 MuSC_WT_Taf4a_1_igg, 37896886, 28070364, 38, paired-end
GSM8533640 MuSC_WT_Taf4a_2, 35536899, 25223271, 38, paired-end
GSM8533641 MuSC_WT_Taf4a_2_igg, 36097123, 24905747, 38, paired-end
GSM8533642 MuSC_WT_Taf4a_3, 27505461, 20062280, 61, paired-end
GSM8533643 MuSC_WT_Taf4a_3_igg, 20294119, 15908972, 61, paired-end
GSM8533644 MuSC_WT_Kansl2_1, 27919402, 19986414, 61, paired-end
GSM8533645 MuSC_WT_Kansl2_1_igg, 20294119, 15908972, 61, paired-end
GSM8533646 MuSC_WT_Kansl2_2, 27194112, 21021755, 61, paired-end
GSM8533647 MuSC_WT_Kansl2_2_igg, 24701440, 15550726, 61, paired-end
GSM8533648 MuSC_WT_Kansl2_3, 29015157, 17950854, 61, paired-end
GSM8533649 MuSC_WT_Kansl2_3_igg, 32433737, 23353309, 61, paired-end
GSM8533650 MuSC_WT_Kansl2_4, 28841214, 21020017, 61, paired-end
GSM8533651 MuSC_WT_Kansl2_4_igg, 27310196, 21212917, 61, paired-end
GSM8533652 MuSC_WT_NF-YA_1, 34553406, 22794692, 38, paired-end
GSM8533653 MuSC_WT_NF-YA_1_igg, 36097123, 24905747, 38, paired-end
GSM8533654 MuSC_WT_NF-YA_2, 52188498, 42182332, 61, paired-end
GSM8533655 MuSC_WT_NF-YA_2_igg, 32433737, 23353309, 61, paired-end
GSM8533656 MuSC_WT_NF-YA_3, 51305858, 41236677, 61, paired-end
GSM8533657 MuSC_WT_NF-YA_3_igg, 27310196, 21212917, 61, paired-end
GSM8533658 MuSC_WT_Nfya_1, 49032037, 25894827, 36, paired-end
GSM8533659 MuSC_WT_Nfya_1_igg, 33657221, 21679573, 36, paired-end

GSM8533660 MuSC_WT_Nfya_2, 32893211, 22605857, 36, paired-end
GSM8533661 MuSC_WT_Nfya_2_igg, 45539328, 8393161, 36, paired-end
GSM8533662 MuSC_Taf4a-KO_Nfya_1, 40911204, 27585732, 36, paired-end
GSM8533663 MuSC_Taf4a-KO_Nfya_1_igg, 42216033, 25172928, 36, paired-end
GSM8533664 MuSC_Taf4a-KO_Nfya_2, 45515562, 33213348, 36, paired-end
GSM8533665 MuSC_Taf4a-KO_Nfya_2_igg, 44369457, 27696251, 36, paired-end
GSM9047391 WT_1, 86049543, 75926702, 36, paired-end
GSM9047392 WT_2, 125511797, 109714419, 36, paired-end
GSM9047393 WT_3, 102555143, 90365343, 36, paired-end
GSM9047394 ko-Taf4a_1, 89321539, 78695261, 36, paired-end
GSM9047395 ko-Taf4a_2, 87178800, 77296356, 36, paired-end
GSM9047396 ko-Taf4a_3, 96111716, 84614924, 36, paired-end

Antibodies

Taf4a (TAFIIp135(22)), Santa Cruz Sc-136093
NF-YA, Santa Cruz Sc-17753
Kansl2, Proteintech 27261-1-AP

Peak calling parameters

Peak calling was performed with either 1) Macs version 2.1.1: macs2 callpeak -t $sample.bam -c $igg.bam -g mm -q 0.05 --extsize 100 -f BAM --nomodel --to-large (GSE277867,GSE277868,GSE277869), or 2) Macs version 3.0.0a7: macs3 callpeak -t $sample.bam -c $igg.bam -g mm -q 0.1 --extsize 75 -f BAM --nomodel --to-large (GSE277870), or 3) Macs version 3.0.0a7: macs3 callpeak -t $sample.bam -g mm -q 0.0001 --extsize 200 -f BAMPE (GSE299832)

Data quality

Raw reads were assessed for quality using FastQC and 100 random reads were compared vs. NCBI NT database to check for contamination. Reads were mapped with STAR and only uniquely mapping, deduplicated, non-spliced reads were used for peak calling. Macs2/3 was run several times with different parameters to find cutoffs that would result in significant peaks. Effects were visually inspected in IGV.

Peaks at <5% FDR and >5x enrichment:
GSM8533638 MuSC_WT_Taf4a_1 423
GSM8533640 MuSC_WT_Taf4a_2 81
GSM8533642 MuSC_WT_Taf4a_3 730
GSM8533644 MuSC_WT_Kansl2_1 28272
GSM8533646 MuSC_WT_Kansl2_2 356
GSM8533648 MuSC_WT_Kansl2_3 597
GSM8533650 MuSC_WT_Kansl2_4 786
GSM8533652 MuSC_WT_NF-YA_1 7960
GSM8533654 MuSC_WT_NF-YA_2 2682
GSM8533656 MuSC_WT_NF-YA_3 3640
GSM8533658 MuSC_WT_Nfya_1 4075
GSM8533660 MuSC_WT_Nfya_2 760
GSM8533662 MuSC_Taf4a-KO_Nfya_1 6932
GSM8533664 MuSC_Taf4a-KO_Nfya_2 2302
GSM9047391 WT_1 55397
GSM9047392 WT_2 64413
GSM9047393 WT_3 64301
GSM9047394 ko-Taf4a_1 60766
GSM9047395 ko-Taf4a_2 57847
GSM9047396 ko-Taf4a_3 62300

Software

STAR 2.7.9a
Picard 2.25.5 (https://broadinstitute.github.io/picard/)
Trimmomatic version 0.39
Macs version 2.1.1-3.0.0a7
DESeq2 version 1.30.0

Flow Cytometry

Plots

Confirm that:

- [x] The axis labels state the marker and fluorochrome used (e.g. CD4-FITC).
- [x] The axis scales are clearly visible. Include numbers along axes only for bottom left plot of group (a 'group' is an analysis of identical markers).
- [x] All plots are contour plots with outliers or pseudocolor plots.
- [x] A numerical value for number of cells or percentage (with statistics) is provided.

Methodology

Sample preparation	Limb and trunk muscles were minced, digested with 100 CU Dispase (BD) and 0.2% type II collagenase (Worthington Biochemicals), and consecutively filtered through 100 µm, 70 µm, and 40 µm cell strainers (BD). Cells were collected by centrifugation at 1,200 x g for 7 minutes. Pellets were re-suspended in 1.5 ml red blood cell lysis buffer containing 5 µg/mL DNase I and incubated on ice for 3 minutes. Subsequently, the cell suspension was filled up to 7 ml with DMEM medium containing 2% FCS, before cells were spun down. To enrich for MuSCs, isolated cells were incubated with APC fluorescence coupled primary antibodies against Sca1, CD45, CD31 (1:100 dilution in FACS sorting buffer) for 40 min at 4oC. After addition of 5ml of DMEM medium containing 2% FCS, cells were spun down and the cell pellets were resuspended in 200µl FACS sorting buffer, before incubation with 30µl of anti-APC micro beads (MACS) for 15 min on 4oC. Microbeads containing Sca1+/CD45+/CD31+ cells were isolated by a 25 LS separation column using the QuadroMACS separator (Miltenyi Biotec). Sca1-/CD45-/CD31- cells were spun down, stained with Integrin-a7-FITC (1:50 dilution in FACS sorting buffer). Integrin-a7+ or GFP+ (from Pax7:ZsGreen mice) satellite cells was isolated using a FACS AriaIII (BD Biosciences).
Instrument	FACS sorting for muscle stem cells: FACSAriaTM III (BD Biosciences)
Software	BD FACS Diva v8 software
Cell population abundance	Sorted cells were reanalyzed to assess purity. A 70-80% purity was achieved.
Gating strategy	The gating strategy identified muscle stem cells by using Integrin-a7-FITC or by taking advantage of endogenous muscle stem cell-specific fluorescence of reporter mice (Pax7:ZsGreen mice). MuSCs population was defined using FMO (Fluorescense Minus One) FITC control (not treated with Integrin a7 – FITC) and dead cells were excluded via DAPI staining. The gating strategy is shown on Supplementary figure 7.

- [x] Tick this box to confirm that a figure exemplifying the gating strategy is provided in the Supplementary Information.
